# Towards low-cost QEPAS sensors for nitrogen dioxide detection

**DOI:** 10.1016/j.pacs.2020.100169

**Published:** 2020-03-10

**Authors:** P. Breitegger, B. Schweighofer, H. Wegleiter, M. Knoll, B. Lang, A. Bergmann

**Affiliations:** Institute of Electrical Measurement and Sensor Systems, Graz University of Technology, 8010 Graz, Austria

**Keywords:** Bare fork quartz-enhanced photoacoustic spectroscopy (QEPAS), Acoustic filters, Quartz tuning fork, NO_2_ detection, Environmental conditions, Drift stability

## Abstract

Increasing awareness of the adverse health effects of air pollution leads to a demand of low-cost sensors for the measurement of pollutants such as NO_2_. However, commercially available low-cost sensors lack accuracy and long-term stability, and suffer from cross-sensitivity to other gases. These drawbacks can be overcome by the method of quartz-enhanced photoacoustic spectroscopy (QEPAS). In QEPAS modulated light is absorbed by the NO_2_ molecules, which results in the production of a sound wave. The sound wave is detected by resonance of a quartz tuning fork, which results in a measurable electric signal. Due to the small size of the tuning forks, the gas sensing element can be smaller than 1 cm^3^. We present the first bare fork QEPAS setup for the ppb-level detection of NO_2_, which is ideally suited for environmental trace gas detection without the need of using micro-resonators. Micro-resonators are commonly used to amplify photoacoustic signals. However, micro-resonators have different dependencies on environmental conditions than tuning forks, which makes them difficult to operate in changing conditions. In contrast, our bare fork QEPAS setup is more robust and easily adopted by the use of a low-cost temperature and humidity sensor. By using acoustic filters the integration time could be increased to offer higher sensitivity at a continuous flow rate of 200 std cm^3^ min^−1^. The 1*σ* noise equivalent concentration is determined to 21 ppb NO_2_ in synthetic air for 120 s measurement time, allowing detection which satisfies international health and safety standards thresholds.

## Highlights

• NO_2_ trace gas detection by means of bare fork quartz enhanced photoacoustic spectroscopy (QEPAS).
• The dependency of the photoacoustic signal on environmental conditions was discussed and investigated.
• Acoustic filters were designed to decrease noise and delay drift to allow longer averaging times.
• A noise analysis was performed.

## Graphical abstract

**Figure:**
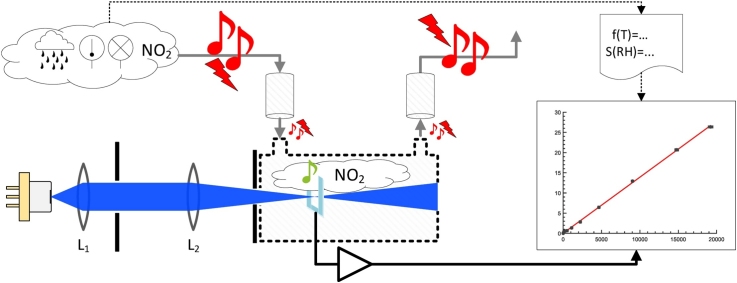

## 1 Introduction

Despite adverse health effects, premature deaths and high costs of air pollution, citizens even in the EU are still exposed to pollutant concentrations exceeding the EU and WHO reference concentrations [1]. Among the pollutants of highest interest is nitrogen dioxide (NO_2_). WHO recommends an hourly mean of 200 μg m^−3^ (106.4 ppb) and an annual mean of 40 μg m^−3^ (21.3 ppb) not to be exceeded [2].

At present, air pollution monitoring is carried out at low spatial resolution due to high costs of highly accurate measurement equipment [3]. To achieve higher spatial resolution, a denser sensor network of low-cost sensors is required. The detection of NO_2_ in sensor networks is usually done by electrochemical-based sensors. These sensors are low-cost but lack long-term stability and suffer from cross sensitivity to other gases [4]. Therefore, our approach for sensing NO_2_ is based on quartz-enhanced photoacoustic spectroscopy (QEPAS) [5] to overcome the aforementioned drawbacks.

Photoacoustic spectroscopy (PAS) uses the effect of sound generation at frequency *f* by modulating a light source at the same frequency. When the wavelength of the light source is chosen such that it matches an absorption line of the analyte, which does not interfere with other analytes in the gas mixture, PAS delivers a sound signal that is directly proportional to the analyte concentration [6]. By using acoustic resonators the signal is further amplified. Recently, a photoacoustic (PA) setup for NO_2_ detection has been validated to sense environmental NO_2_ concentrations with good agreement to an environmental monitoring station [7]. However, conventional PAS setups have relatively large footprints because the detection bandwidth of available microphones is limited to several kHz, which further leads to acoustic resonators in the cm range. For example, the size of the 1.75 kHz PA resonator in Yin et al. [7] was approximately 120 mm × 40 mm × 40 mm. However, sensor sizes of approximately 50 mm × 50 mm × 30 mm [8] are favored for environmental sensors, as size usually scales with cost.

In contrast to conventional PAS, QEPAS applies piezoelectric quartz tuning forks (QTFs) as acoustic transducers. The transducers usually have resonance frequencies around 32.768 kHz which correspond to acoustic wavelengths of approximately 10.5 mm with a typical length of the QTF's prongs being 3.8 mm. In addition, since QTFs are mechanical resonators, they have quality (*Q*) factors greater than 8000 which provides excellent amplification. In contrast, the Q factor of the setup by Yin et al. [7] is 25. As piezoelectric QTFs are mass produced for the use as clocks in quartz watches they are available for prices in the cent range. Additionally, QEPAS setups offer high background noise immunity, e.g. QEPAS yielded an improvement in noise immunity by a factor of 46 compared to a conventional PAS setup in traffic noise simulations [9].

For the detection of NO_2_, a number of low-cost light sources in the visible (VIS) range of light are available. Due to the broad absorption spectrum of NO_2_, in the visible range, however without pronounced absorption peaks, modulation of the light source has always be done by amplitude modulation. QEPAS setups for NO_2_ detection usually rely on light sources with a peak emission around 450 nm. At this wavelength, NO_2_ yields high absorption and photodissociation of NO_2_ is not promoted [10].

**Fig. 1:**
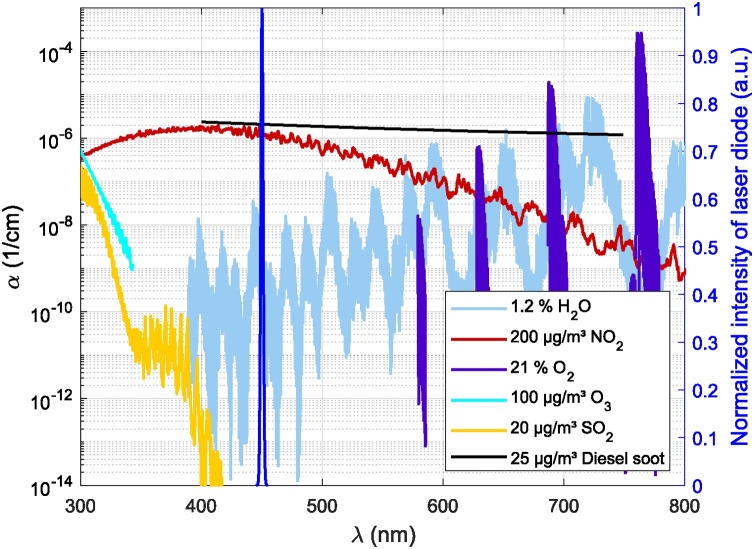
Absorption of different environmental gases between 300 and 800 nm according to the HITRAN database [12], [13] and absorption of diesel soot between 400 and 750 nm [14]. Concentrations reflect the current WHO recommended limits (NO_2_: 1-h mean, O_3_: 8-h mean, SO_2_: 24-h mean, Diesel soot (PM_2.5_): 24-h mean) [15]. Laser emission spectrum is shown in blue.

Further, this wavelength yields negligible cross-interference to other gases (cf. Fig. 1). To overcome possible cross-interference by the broad absorption range of soot (cf. [11]) a filter can be placed at the sampling inlet.

To date QEPAS sensors for NO_2_ have relied on off-beam (ob) configuration in which the laser is directed through a micro-resonator, with the first longitudinal mode sharing the same resonance frequency as the QTF. A small slit in the middle of the micro-resonator is directed between the QTF's prongs, which allows the QTF to sense the amplified acoustic wave. This configuration has two advantages: First, the acoustic signal is additionally amplified by a factor of 15 as compared to bare QTF setups [16]. Second, the beam quality can be worse than for bare QTF setups, due to the larger diameter of the micro-resonator (e.g. 1.6 mm in Rück et al. [9]) as compared to the distance between the prongs of commercial QTFs (approximately 300 μm in this publication). However, ob-QEPAS has one disadvantage, especially relevant for ambient measurements: The micro-resonator and QTF are affected differently by environmental conditions such as temperature, pressure and gas composition. Therefore, the resonance frequency of the two resonators shift differently with changing conditions, resulting in huge variations of the combined amplification. This detuning effect was seen by Rück et al. [9], where the sensor signal rose by 35% when increasing relative humidity from 0% to 80%.

A limit of detection of at least 106.4 ppb NO_2_ is required to detect the exceedance of hourly mean thresholds, which was achieved by the previous QEPAS setups. Detection limits of 600 ppt NO_2_ [9], 1.3 ppb NO_2_ [17], and 4.4 ppb NO_2_ [18] are reported for ob-QEPAS configurations. However, ob- configurations suffer from their high dependency on environmental conditions.

Therefore, we employ bare fork (bf) QEPAS for detection of NO_2_ to demonstrate limits of detection appropriate for environmental monitoring. In this configuration, the *Q* factor is insensitive to temperature, pressure and changes of gas composition when compared with off-beam methods, thereby providing opportunity for corrections of the resonance frequency. This will be highlighted by discussing models for the tuning fork and micro-resonators. The integration time could be increased to previously unattained values by the use of acoustic filters, which also reduce gas flow noise.

## 2 Material and methods

**Fig. 2:**
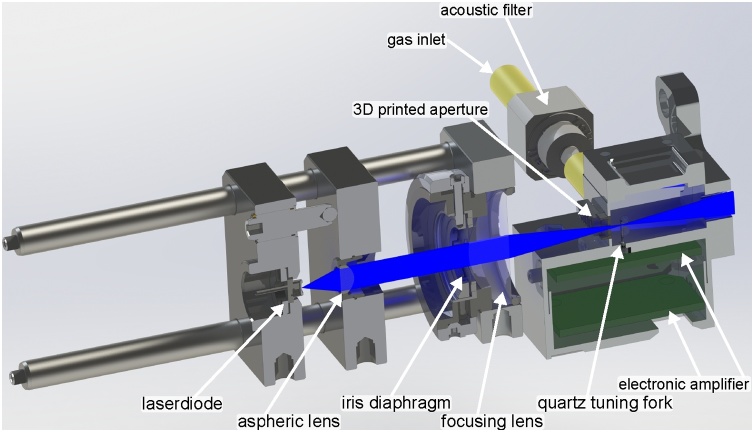
Cut through the optical setup and the 3D-printed cell. Laser beam is shown in blue. The gas outlet is perpendicular to the inlet. Description of the elements can be found in the text. The distance from the laser diode mount to the back of the cell is ≈10 cm.

A cut through the cell and focusing optics is shown in Fig. 2. A laser diode (OSRAM: PL 450B) is used as excitation light source. The laser diode mount passively cooles the laser diode. The collimation and focusing optics is mounted in a cage system. The laser diode is collimated by an aspheric lens (Thorlabs: A220TM-A) and stray-light is removed from the collimated beam with an iris diaphragm, which is set to approximately 900 μm. The beam is focused into the gas cell and between the prongs of the QTF (Fox Electronics: NC38LF) with a *f* = 30 mm focusing lens. To remove stray-light more effectively, a 3D printed aperture of approximately 500 μm is placed at the front of the cell. The focus of the laser beam, which is of 116 μm width (cf. Appendix A), is adjusted to pass the prongs of the QTF approximately 0.7 mm from the top of the prongs for a strong signal (cf. [5]). The 3D printed gas cell has a sample volume of 20.6 mm × 16 mm × 9.7 mm, which can easily be miniaturized to smaller dimensions. The cell, which also carries the two stage amplifier, is covered by aluminum tape, which is grounded for electronic shielding. An unshielded version of the cell resulted in signal fluctuations corresponding to ppm concentrations of NO_2_. All 3D printed parts are printed with a stereolithographic 3D printer (FORMLABS: Form 2) with 25 μm resolution.

**Fig. 3:**
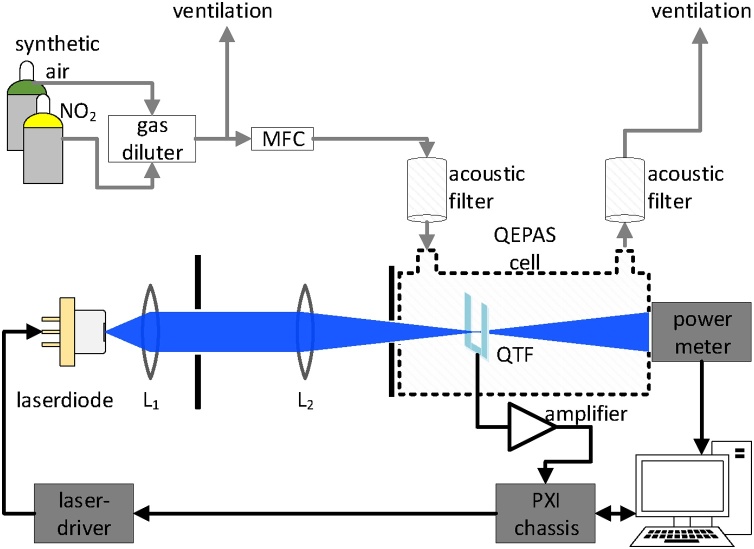
Schematic of the experimental setup for characterizing the response to NO_2_. L_1_, aspheric lens; L_2_, focusing lens; QTF, quartz tuning fork.

The experimental setup is shown in Fig. 3. Gas mixtures are passed to the QEPAS cell at a flow rate of 200 std cm^3^ min^−1^, controlled by a mass flow controller (MFC; Vögtlin: Model GSC-B). Acoustic filters at the in- and outlet of the cell improve noise and drift characteristics (cf. Section 4.2).

The signal from the QTF is amplified with a custom two-stage amplifier. The signal is acquired with a data acquisition (DAQ) card (National Instruments: Model PXI-6281) at 250 kSps and post-processed on a personal computer (PC). The same chassis carrying the PXI-6281 also houses a function generator (National Instruments: Model PXI-5402). The function generator provides the square wave modulation signal (50% duty cycle) for the laser driver (Thorlabs: ITC4001). The laser modulation results in an average optical power of approximately 46.9 mW (±3% at 450 nm), measured by means of a power meter (Thorlabs: Model S120C). This is slightly higher than the expected half nominal cw optical power of 40 mW.

The amplified QTF-signal is filtered with a lock-in amplifier, which is realized as a custom LabVIEW application on a PC. The integration time is set to 1 s. Due to the limited buffer size of the DAQ card, data acquisition and modulation is stopped and restarted after each measurement to obtain the same phase. Since the QTF is excited from zero at each measurement, the buildup phase and release time, which result from the high Q factor of the resonator have to be removed from the measurement. The timescale can be calculated as *τ* = *Q*/*πf*_0_ ≈ 100 ms. To reduce uncertainties in the measurement, the first 0.5 s of the measurement are removed and therefore, the measurement time is extended by 0.5 s compared to the integration time.

All measurements were performed at atmospheric pressure. Gas mixtures were produced with a temperature stabilized custom gas diluter based on binary weighted critical orifices [19], which offers low uncertainties at high dilution ratios. The NO_2_ gas cylinder contains a mixture of NO_2_ and synthetic air (Messer Austria GmbH: 19.2 ppm NO_2_). This mixture was further diluted with synthetic air (Messer Austria GmbH: Synthetic Air, Scientific).

## 3 Influence of gas composition on the photoacoustic signal

Effects of different environmental conditions must be considered during calibration of QEPAS sensors for ambient measurements. Changes in environmental conditions affect the resonance frequency and the *Q*-factor of a QTF. E.g. for *Q* = 8000, a resonance frequency shift of 1 Hz results in a 10% signal drop. As will be shown, temperature has the biggest influence on the QTF by affecting its resonance frequency. Expected pressure changes in environmental sensing only have a minor effect on resonance frequency and *Q*-factor. Humidity promotes a faster relaxation of the excited states, resulting in a humidity-enhanced signal [6]. An excitation frequency adjustment based on a temperature sensor and a signal adjustment based on a humidity sensor can be implemented in a bf-QEPAS setup.

In contrast, ob-QEPAS setups strongly depend on temperature, humidity and the speed of sound of the gas mixture, which also changes with humidity [9], [20]. As the resonance frequencies of the micro-resonator and QTF shift differently with changing conditions, the combined *Q*-factor varies greatly and cannot easily be described, making ob-QEPAS less suitable for low-cost environmental sensors than bf-QEPAS. This is summarized in Table 1 and shown in the next sections, e.g. by considering typically expected variations of temperature and pressure in environmental sensing.

**Table 1:** Summary of the influences of temperature (*T*), pressure (*p*), and humidity (*H*) of the resonance frequency *f*_0_, the quality factor *Q* and the photoacoustic signal *S*. The sign before the slash defines how much the quantity is affected (+ – not, o – little, - – much), the second defines how easy a correction can be done (+ – easy, o – with effort, - – not possible), e.g. for bf-QEPAS, the temperature influence on the resonance frequency *f*_0_ is little (o) and can easily be corrected (+).

	Bare fork			Off-beam		
	*Q*	*f*_0_	*S*	*Q*	*f*_0_	*S*
*T*	+/n.a.	o/+	o/+	-/o	-/o	o/+
*p*	o/+	o/+	+/n.a.	o/+	o/+	+/n.a.
*H*	+/n.a.	+/n.a.	-/+	-/-	-/-	-/-

### 3.1 Temperature

**Fig. 4:**
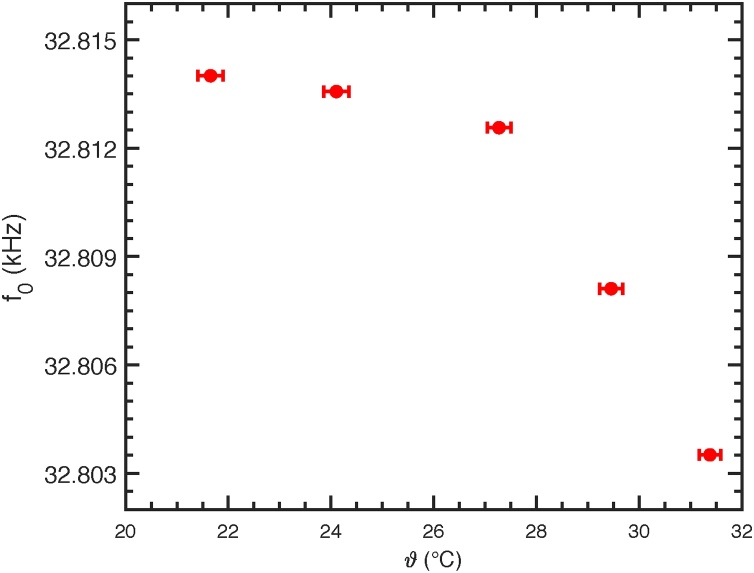
Temperature dependence of the resonance frequency.

To investigate the temperature dependence of a QTF's resonance frequency, heated gas was applied to the bf-QEPAS sensor. The temperature was measured by means of a temperature sensor (Sensirion AG: SHT31), which was mounted into the cap of the bf-QEPAS sensor. After the temperature in the cell remained at a stable value, a series of eight sweeps was performed with synthetic air and 19.2 ppm NO_2_, respectively. The resonance frequencies were determined by fits to these sweeps and are depicted in Fig. 4. The temperature dependence is evident and justifies that the resonance frequency should be determined after a change in temperature is detected. The QTF used for this work is specified for operation between −20 and 60 °C [21], but temperatures below and above this range are also possible for quartz [22]. Since the noise in QEPAS is proportional to $\sqrt{T}$ (cf. Eq. (5)), the sensitivity is assumed to improve with decreasing temperature.

**Fig. 5:**
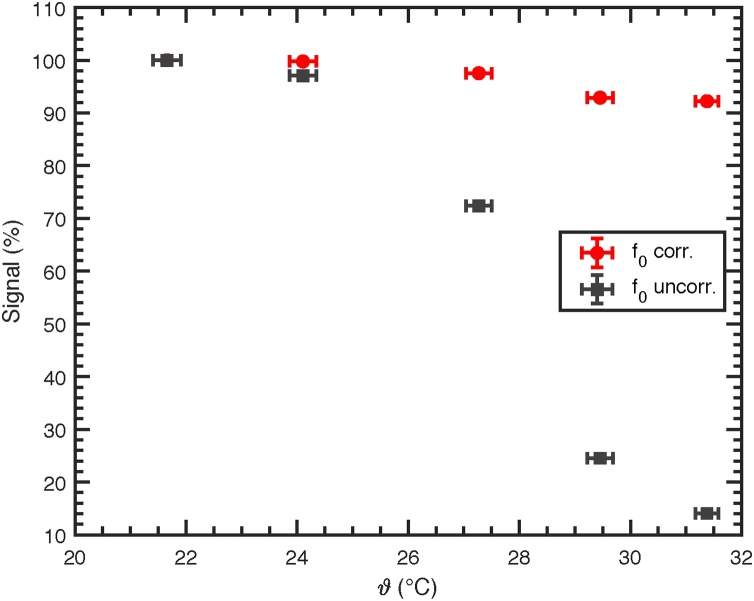
Measured signal with 19.2 ppm NO_2_ in % of the signal measured at 21.6 °C as function of temperature. Signal with frequency adoption (red); Signal without frequency adoption (black).

Fig. 5 displays the background corrected signal with NO_2_. The signal decreases to 14% of the original signal if the resonance frequency is not adopted after a temperature increase of 10 °C. In contrast, by applying a resonance correction, the signal has a maximum deviation of 8%. Deviations from 100% are caused by the 1/*c*^2^ ∝ 1/*T* dependence of the QEPAS pressure [23], which can easily be corrected by taking into account the actual temperature of the gas. A dependence of the *Q* factor on temperature could not be observed.

However, in ob-QEPAS micro-resonators are used to acoustically amplify the PA signal. Micro-resonators are open ended acoustic resonators. The resonance frequency *f*_*mr*_ of the fundamental longitudinal oscillation is given by [9]

(1) $$f_{\mathrm{mr}}=\frac{c_{s}}{2L_{\mathrm{res}}},$$

where *L*_*res*_ is the resonator length and *c*_*s*_ the speed of sound. According to the ideal gas law, *c*_*s*_ depends on the temperature *T* as $\sqrt{T}$. Therefore the resonance frequency of QTF and micro-resonator shift contrary with respect to *T*. A temperature change from 25 to 35 °C increases the micro-resonator's resonance frequency by roughly 550 Hz, which shifts the resonance curve with respect to that of the QTF, where the resonance frequency would decrease by roughly 10 Hz, resulting in a tremendous change of the combined *Q*-factor and thus the signal strength. E.g. a doubling of the combined *Q*-factor of an ob-QEPAS setup by a temperature increase from approximately 315 to 345 K is reported by Köhring et al. [20] – an issue which is avoided in the present bf-QEPAS setup.

### 3.2 Pressure

A pressure change influences the resonance frequency and the *Q*-factor of the QTF. Kosterev et al. [24] described the dependence of the QTF's resonance frequency on the pressure, *f*_*res*_(*p*), by

(2) $$f_{\mathrm{res}}(p)=f_{\mathrm{vac}}-\frac{{\mathrm{df}}_{\mathrm{res}}}{\mathrm{dp}}p,$$

where *f*_*vac*_ is the resonance frequency in vacuum and $\frac{{\mathrm{df}}_{\mathrm{res}}}{\mathrm{dp}}=7.2\times 10^{-3}$ Hz mbar^−1^. For a pressure change from 970 to 1013.25 mbar, the resonance frequency would change by 0.3 Hz, resulting in a minor signal drop of 1% if not corrected. The pressure dependence of the *Q*-factor of a QTF of the same dimensions as it was used for this publication can be described in form of [24]

(3) $$Q(p)=\frac{Q_{\mathrm{vac}}}{1+Q_{\mathrm{vac}}ap^{b}},$$

where *a* = 2.8 × 10^−6^ and *b* = 0.47. For the same pressure change as above, *Q* would decrease by 2%, resulting in a signal drop of 2%. However, as both expressions are known, corrections can easily be implemented in bf-QEPAS.

To study the dependence of a micro-resonator's resonance frequency on pressure, the speed of sound in Eq. (1) can be rewritten by using the ideal gas law to $c_{s}=\sqrt{\gamma \frac{p}{\rho }}$, with *p* the pressure, *ρ* the density and *γ* the adiabatic index. As *p* and *ρ* are directly proportional via the ideal gas law, the resonance frequency of the micro-resonator is independent of the pressure.

### 3.3 Humidity

The dependence *η* of the photoacoustic signal on the relaxation time *τ* and the modulation frequency *f*_0_ from the exited states can be described by Eq. (4) [6]:

(4) $$\eta =\frac{1}{\sqrt{1+{\left(2\pi f_{0}\tau \right)}^{2}}}.$$

For NO_2_, *τ* < 4 μs is reported [25], which already affects the signal strength for the modulation frequency of *f*_0_ ≈ 32.8 kHz. Adding water vapor has been shown to speeding up the delayed molecular relaxation also for NO_2_ and modulation frequencies of 1.75 kHz [7].

**Fig. 6:**
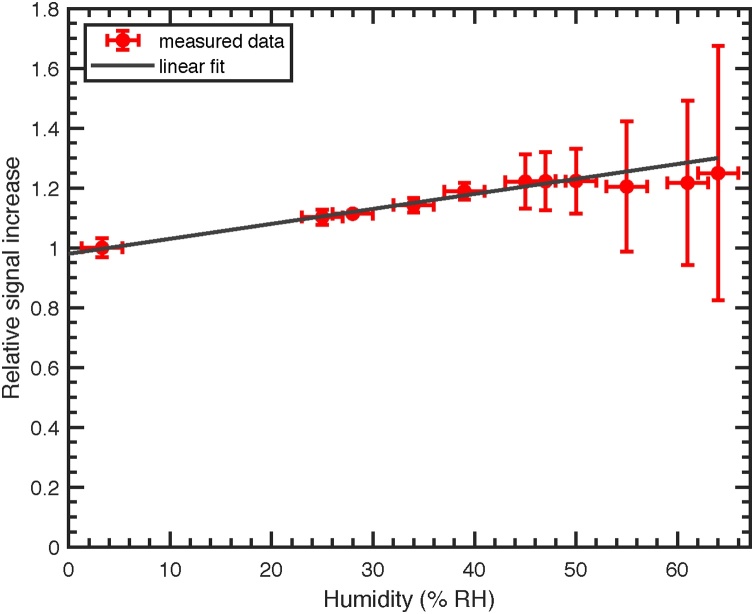
Relative QEPAS signal with respect to the humidity in the reference gas cylinder as a function of relative humidity (red) and linear fit (black). Errorbars at high humidities result from the high uncertainty at low flow rates of the MFCs used.

The effect of humidity on the photoacoustic signal is analyzed by applying different mixtures of NO_2_ and humidified air to the QEPAS cell. In order to prevent a reaction between NO_2_ and H_2_O, NO_2_ was added after the humidifier. The concentrations were adjusted with two MFCs (Vgtlin: Model GSC-B), which were calibrated by bubble flow meters (Gilian: Gilibrator 2). Relative humidity was measured by means of a humidity sensor (Sensirion: SHT31). Relative humidity ranged from 3.3% RH (water concentration of 0.08%) to 64% RH (water concentration of 1.52%). The temperature in the cell was 24 °C. Sensor signals are normalized by the corresponding NO_2_ concentrations and referenced to the 3.3% RH humidity measurement (19.2 ppm NO_2_ from the gas cylinder). The corresponding plot is depicted in Fig. 6, where a signal enhancement, due to added humidity can clearly be seen. An improvement of uncertainty at high humidities could be achieved by using MFCs calibrated for lower flow rates.

The increase might be approximated by *S*_*N*_ = *a* · *x* + *b*, where *S*_*N*_ is the normalized QEPAS signal, *x* the relative humidity concentration in percent, and *a* = 0.98 and *b* = 0.005 are fit parameters. This signal enhancement is likely to be caused by a drop in relaxation time of the excited state as seen for other molecules such as CO_2_, HCN and CH_4_ for added humidity [26], [27], [28].

Finally, no changes of resonance frequency or quality factor were found. Thus, the sensor reading of the bf-QEPAS sensor can easily be corrected by the above equation and the use of a humidity sensor reading. In contrast, signal enhancement due to humidity in an ob-QEPAS setup leads to a detuning between QTF and micro resonator [9]. This is caused by the fact that the resonance frequency of a micro resonator is depending on the speed of sound (cf. Eq. (1)), which depends on the adiabatic index as $\sqrt{\gamma }$. In contrast, the resonance frequency of the QTF is independent of the speed of sound. Thus, the combination of the two effects does not allow for straight-forward signal corrections in ob-QEPAS.

## 4 Noise analysis

To investigate the individual noise contributions and possible improvements of the setup, the fundamental noise of the QTF is investigated. Then, Allan deviation is calculated to investigate sources of noise and drift.

### 4.1 Thermal noise and amplification topology

**Fig. 7:**
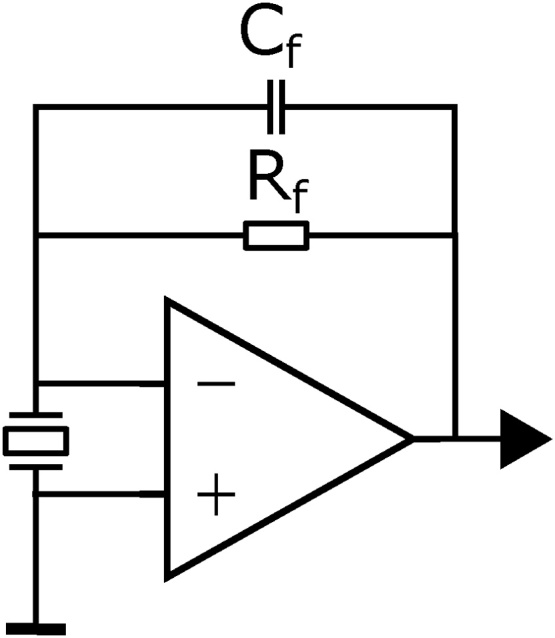
Schematic drawing of the TIA topology used for this work.

The noise in QEPAS is fundamentally limited by the thermal noise of the QTF. To investigate the thermal noise, the electronic amplification circuit must be characterized first. In this work, the QTF signal was amplified by a custom circuit consisting of a transimpedance amplifier (TIA) and a non-inverting amplifier with an amplification of 270. The TIA topology (Fig. 7) uses a *LTC6240HV* low noise operational amplifier with a feedback resistor of *R*_*f*_ = 6.8 MΩ with a 0.3 pF capacitor in parallel for stability reasons.

**Fig. 8:**
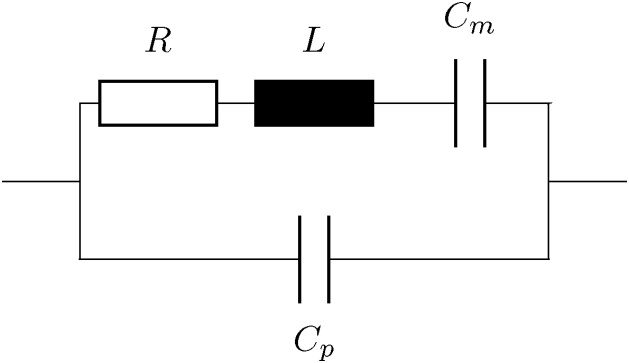
Butterworth–Van Dyke equivalence circuit of a QTF.

As discussed by Starecki and Wieczorek [29], a TIA behaves like a low-pass at high frequencies. For the given configuration, stray and other capacitances *C*_*s*_ are estimated to be 1.5 pF. This results in a cut-off frequency of $f_{\mathrm{co}}=\frac{1}{2\pi R_{f}C_{s}}\approx 16$ kHz. The corresponding amplification at the resonance frequency is therefore roughly 43% of its value at low frequencies. Since noise is attenuated by the same factor, the signal-to-noise ratio remains unchanged but it needs to be considered by performing the consecutive calculations with a reduced feedback resistor $R_{f}^{\prime }=R_{f}\cdot 0.43\approx 2.9$ MΩ. This behavior is important and usually neglected for estimating the thermal noise of the QTF. The thermal noise of the QTF also depends on the resistance of its Butterworth–Van Dyke equivalence circuit [30] as shown in Fig. 8. Here, *R* is the resistance, *L* is the inductance, *C*_*m*_ is the motional capacitance, and *C*_*p*_ is the parasitic capacitance.

The thermal noise in the TIA topology, $\sqrt{<V_{N}^{2}>}$, is given by [31]

(5) $$\sqrt{\langle V_{N}^{2}\rangle }=R_{f}^{\prime }\sqrt{\frac{\Delta f4k_{B}T}{R}}.$$

In this equation, $R_{f}^{\prime }$ is the reduced feedback resistor, Δ*f* is the detection bandwidth, *k*_*B*_ is the Boltzmann constant, and *T* is the temperature. It is therefore crucial to determine *R* to evaluate the fundamental noise floor.

In this work, *R* was determined by thermally exciting the QTF with the laser and fitting the real- and imaginary part of the response signal to the admittance as described by the electric equivalence circuit (Appendix B). The resistance *R* was determined to 85.7 kΩ, resulting in a thermal noise after the TIA of 1.29 μV.

**Fig. B.14:**
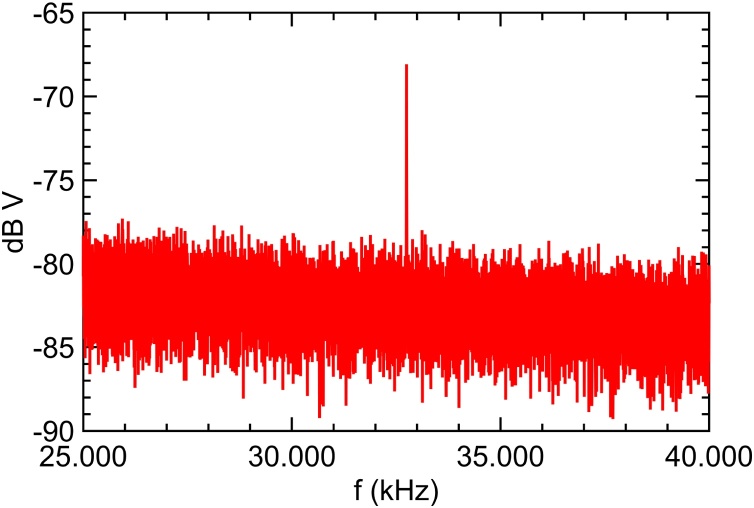
FFT of the average noise floor of the QTF after the second amplifier (factor of 270 higher than the value after the transimpedance amplifier) with the laser switched off. The noise peak at the resonance frequency is −68 dBV, equivalent to 0.4 mV.

The actual noise of the circuit was measured by investigating the average noise of the QTF signal at the resonance frequency with the laser switched off (Fig. B.14). There, the noise after the TIA equates to 1.48 μV. Considering the manufacturing uncertainties of the electric components, the noise of the operational amplifier, as well as the estimation of the stray capacitances, the electronic noise of the circuit is close to its optimum value, leaving little space for improvement of the TIA.

### 4.2 Contribution of noise sources

**Fig. 9:**
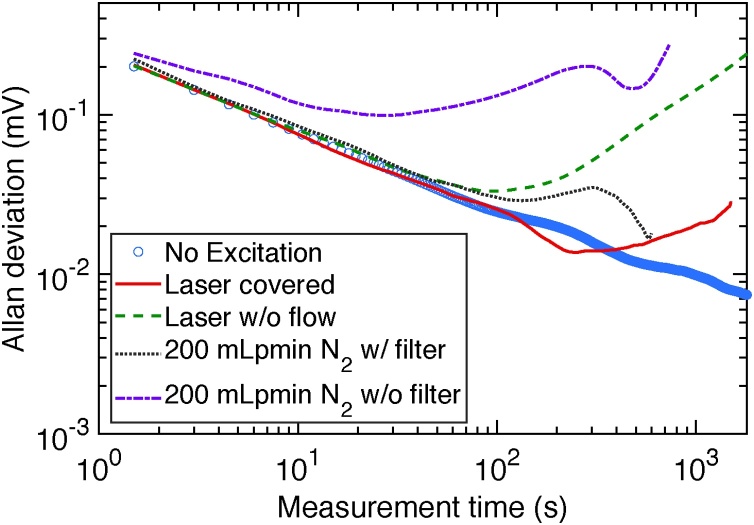
Allan deviation of the photoacoustic signal at different configurations as a function of the measurement time. Allan deviation was calculated with MATLAB (Author: E. Ogier, Version: 1.0, Release: 28th March 2016, MATLAB, calculation: classical type, Title: AVAR).

Allan deviation was calculated to investigate sources of noise and drift for different configurations. In Fig. 9, Allan deviation is plotted as a function of the measurement time. The integration of the acquired data is simply performed by averaging the values over the measurement period. One data measurement cycle for 1 s averaging time is 1.5 s, due to the previously mentioned 0.5 s startup time. In the Allan deviation plots, the measurement cycles (1.5 s) are simply strung together to give the measurement time. Consequently, using a certain averaging time is supposed to yield even a lower noise than the corresponding Allan deviation in Fig. 9 suggests. To give an example, an averaging time of 10.5 s corresponds to the Allan deviation of 15 s in Fig. 9. This is due to the fact that the 0.5 s startup of the QFT is required only once for a specific averaging time.

The fundamental deviation due to thermal noise (blue) is 201 μV for 1 s averaging time (1.5 s measurement time) and continuously decreasing with increasing averaging time without a drift being visible within the highest averaging time.

**Fig. C.15:**
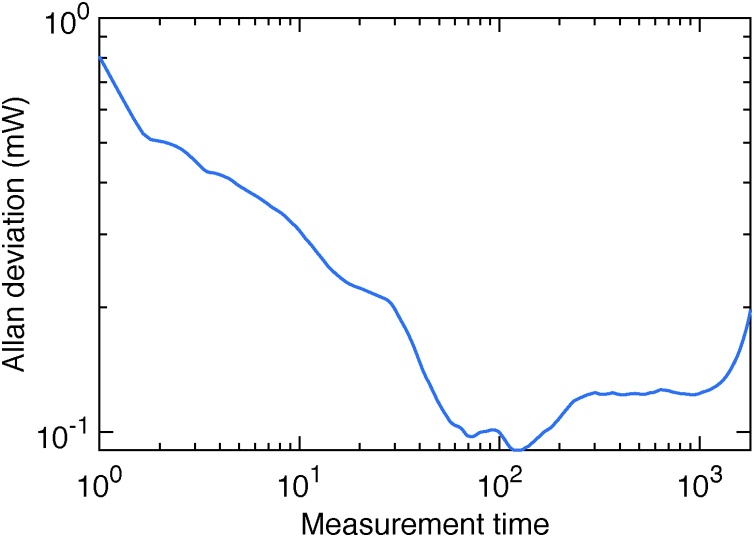
Allan deviation of the laserpower as a function of the averaging time.

Neither switching the covered laser on (red), nor uncovering the laser (green) significantly increases the deviation. However, drift occurs after 225 s measurement time for the covered laser and decreases to 120 s for the uncovered laser due to a drift of the laser power (cf. Fig. C.15).

Flow noise, due to the gas flow (purple) would increase the deviation to 242 μV for 1 s averaging time and the maximum averaging time would be limited to 24 s due to drift. In contrast, due to the acoustic filters (black) the 1 s deviation is reduced to 223 μV and drift is improved to the level determined by the laser drift (120 s measurement time), yielding a value of 29 μV for the corresponding averaging time of 80 s. This allows for much longer averaging times as compared to other QEPAS setups for NO_2_ (20 s [18] and 10 s [9]), which allows to achieve low limits of detection by increasing the integration time.

#### 4.2.1 Acoustic filter design

Due to its high resonance frequency and quadrupole characteristics, QEPAS is little susceptible to external noise [9]. However, to additionally suppress flow noise, noise from outside the cell, and diminish drift, a two stage acoustic filter was designed and 3D printed. Two two-stage filters are used in the setup, one at the inlet and one at the outlet.

**Fig. 10:**
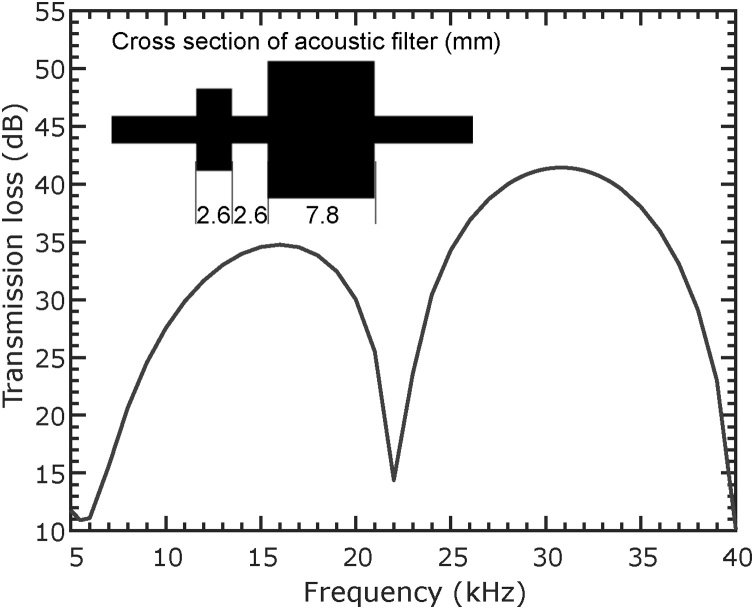
Transmission loss of the two stage acoustic filter. Inset shows the cross section and dimensions of the acoustic filter.

The two filters are expansion chamber mufflers of length 1/4 *λ*_0_ (2.6 mm) and 3/4 *λ*_0_ (7.8 mm), where $\lambda_{0}=\frac{c_{0}}{f_{0}}$ is the wavelength corresponding to the resonance frequency *f*_0_ of the QTF. Diameters are chosen such that only plane waves can propagate (6 and 10 mm, respectively). The combined transmission loss, calculated with the help of COMSOL Multiphysics, is plotted in Fig. 10. As can be seen, the transmission loss is better than 38dB in the range of the resonance frequency.

Further, an eigenfrequency analysis and a frequency sweep over the expected modulation frequency range from 32 to 33.6 kHz was carried out to design the cell such that no unwanted constructive or destructive interferences appear within the cell.

## 5 Linearity, stability and limit of detection

**Fig. 11:**
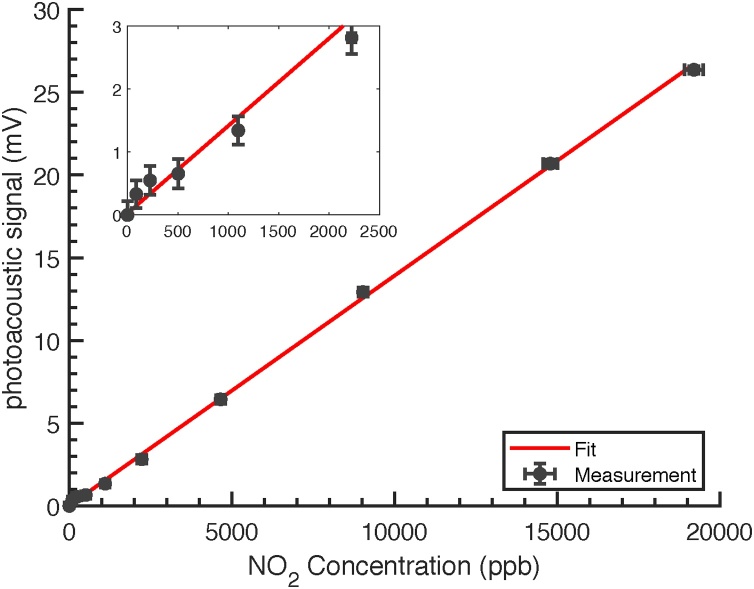
Linear fit (red) of the background corrected photoacoustic signal as a function of the NO_2_ concentration. Errorbars of the PA signals are the standard deviation of the recorded values. Errorbars of the concentrations show the standard deviation calculated with GUM Workbench. The linear curve fit was conducted in MATLAB (Author: Travis Wiens, Version: 1.0.0.0, Release: 2010, Title: Linear Regression with Errors in X and Y).

Fig. 11 shows the QEPAS signal as a function of the NO_2_ concentration which was varied between 87 ppb and 19.2 ppm. Each datapoint was averaged for approximately 80 values. All signals are background corrected with respect to their phase. The linear regression for the photoacoustic signal *S* as a function of concentration *c* equates to *S* = *b* · *c* + *a*, where the slope *b* = (1.39 ± 0.02) μV ppb^−1^ and intercept *a* = (26 ± 95) μV. The coefficient of determination for the fit is *R*^2^ = 0.999.

By using the standard deviation of the noise with acoustic filters from the previous chapter, the noise equivalent concentration (NEC) for 1*σ* and 1 s integration time is determined to 160ppb NO_2_ in synthetic air. This is a factor of 7.7 higher than in [18], when normalizing their value to synthetic air as described in the previous chapter. For the longest possible measurement time, according to the noise analysis, we achieve a normalized NEC of 21 ppb for 120 s which is a feasible value for environmental trace gas detection, since the typical averaging time in environmental monitoring is 1 h [3].

## 6 Discussion

Compared to off-beam configurations, the present system is more robust to changes of environmental conditions such as humidity and temperature. Temperature changes can be taken into account by adjusting the laser modulation frequency and a simple model. Corrections for humidity influences are possible by using a simplified model, taking into account only a humidity sensor reading. Hence, with the simple bf-QEPAS setup and simple models, the overall measurement uncertainty can be reduced significantly making it suitable for environmental trace gas measurements.

In contrast to ob-QEPAS, where production tolerances of micro-resonators would make calibration in a wide parameter space, spanned by temperature, humidity and NO_2_ concentration necessary, simply one linearity curve is sufficient in bf-QEPAS. The dependence of ob-QEPAS on temperature is difficult to compensate due to the different dependencies of micro-resonator and the QTF. A rising temperature leads to a smaller resonance frequency of the micro-resonator, while the resonance frequency of the QTF shows a parabolic behavior with respect to the temperature. Thus, for ob-QEPAS a sample gas conditioning system is necessary to overcome these influences at the cost of size, power consumption and price, which are undesirable in low-cost sensing.

Due to its simple setup, bf-QEPAS has the potential to be mass-produced. In an automated process, the optimal focus point between the prongs can be found with a power meter or a beam profiler positioned at the back of the cell in bf-QEPAS. In contrast, ob-QEPAS relies on positioning of a collimated beam to the microresonator, but also on precise positioning of the micro-resonator to the QTF. It is expected that with little engineering effort, a miniaturization of a bf-configuration to a size of approximately 50 mm × 50mm × 30 mm seems feasible.

## 7 Conclusion and outlook

The presented QEPAS setup is able to reach a 1*σ* detection limit of 21 ppb NO_2_ in synthetic air for 120 s measurement time.

By using the Butterworth–Van Dyke equivalent model, the noise of the preamplifier was determined to be close to the fundamental thermal noise level. The noise level due to acoustic noise could be reduced by using acoustic filters. The use of such acoustic filters is also expected to be advantageous for environmental trace gas sensing, where traffic noise is likely to have a higher contribution than the flow noise. Further, the use of acoustic filters minimizes drift, which allows for measurement times up to 120 s, thereby enabling low detection limits at relatively low laser powers.

In future, the lock-in amplifier as well as the laser driver will be replaced by a low-cost, custom developed electronic solution built around a micro-controller to have a complete low-cost NO_2_ sensor platform. By overcoming the limitation of limited continuous measurements of the present setup, the averaging time can be increased to 120 s, to reach even lower limits of detection. Further, it is assumed that drift of the laser diode can be compensated in software, by monitoring the temperature, the power, or the current of the laser diode. In addition, a mechanical coupling between the optical components and the QTF is planned, to provide stability against external vibrations. To enable lower detection limits at low timescales, the laser diode could be operated in pulsed mode (cf. [32]), as pulsed operation produces a *π*/2 times higher photoacoustic signal [33].

## Conflict of interest

The authors declare no conflict of interest.

## Figures and Tables

**Fig. A.12:**
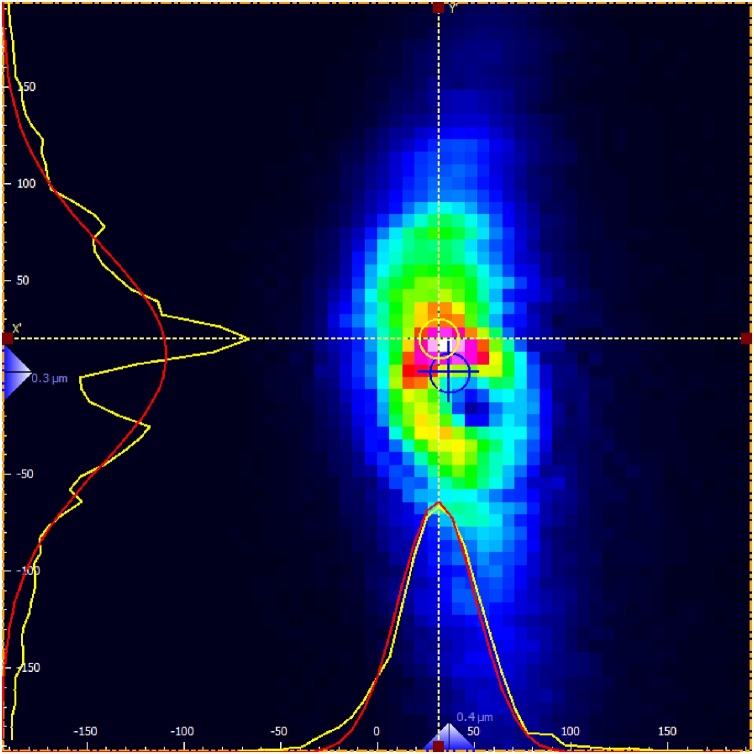
2D projection of the focused laser spot.

**Fig. B.13:**
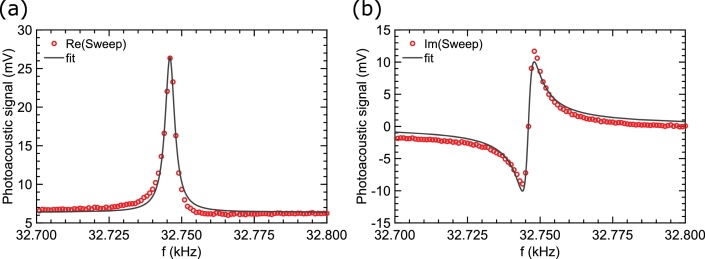
Real (a) and imaginary (b) parts of the photoacoustic signal (black), proportional to the real and imaginary part of the admittance (fit in red).

## Appendix A Beam profile of the focused beam

The 2D projection of the focused laser spot was measured by means of a beam profiler (Thorlabs: BC106N-VIS/M; Fig. A.12). The horizontal and vertical ellipse diameters are 116 and 282 μm, respectively.

## Appendix B Determination of the thermal noise

A QTF can be electrically modelled by a Butterworth-Van Dyke circuit (Fig. 8) [30]. To determine the resistance *R*, we proceeded as follows: (i) de-focus the laser to hit the prongs and thermally excite the QTF, which produces a current, proportional to the admittance of the QTF (ii) sweep through the frequencies of interest, and fit the imaginary and real part of the response signal to the imaginary and real part of the admittance of the Butterworth–Van Dyke circuit, (iii) as the driving voltage, which equals to the thermal excitation is not known, the capacitance *C*_*p*_ is measured, and (iv) inserted as a scaling factor to determine all relevant values.

The real and imaginary parts of the admittance *Y* are given by Eqs. (B.1) and (B.2). In addition to the equations from [30], a parallel admittance *Y*_*p*_ was added to Eq. (B.1) for baseline correction:

(B.1) Re(Y)=Yp+1R·ω2·ωm2(Q·(ω2−ωm2))2+ω2·ωm2

(B.2) Im(Y)=ω·Cp·ω4−ω2·(ωp2+ωm2·(1−1/Q2))+ωm2·ωp2((ω2−ωm2)2+ω2·ωm2/Q2)

In these equations, *ω* = 2*πf*, $\omega_{m}=\frac{1}{\sqrt{LC_{m}}}$, $\omega_{p}=\frac{1}{\sqrt{L\frac{C_{m}C_{p}}{C_{m}+C_{p}}}}$, and $Q=\frac{\omega_{m}L}{R}$.

The corresponding measurements display the real- and imaginary part of the photoacoustic signal, which is proportional to the admittance. Corresponding fits are displayed in Fig. B.13.

The capacitance *C*_*p*_ was measured from an open QTF of the same model by means of a precision LCR Meter (KEYSIGHT E4980AL), resulting in a value of 1.86 pF.

From *ω*_*p*_ and *ω*_*m*_ of the fits, *C*_*m*_ is calculated via $C_{m}=({\left(\frac{\omega_{p}}{\omega_{m}}\right)}^{2}-1)\cdot C_{p}$. By knowing *C*_*m*_, *L* is calculated from *ω*_*m*_. Finally, *R* is calculated from *ω*_*m*_, *L*, and *Q* as 85.7 kΩ.

To compare the theoretical noise to the actual noise of the circuit, the average noise spectrum of the QTF signal with the laser switched off was investigated (Fig. B.14). As the signal was acquired after the second amplifier, it has to be divided by the amplification of the second amplifier, resulting in a noise of 1.48 μV.

## Appendix C Drift due to the laserpower

To investigate whether drift is dominated by fluctuations of the laser power, an Allan deviation analysis, based on the same time resolution as the noise analysis, was carried out. As can be seen in Fig. C.15, a drift of the laserpower is appearing at the same timescale (around 120 s), which indicates that drift is caused by fluctuations of the laserpower.
